# Venous Thromboembolism in an Industrial North American City: Temporal Distribution and Association with Particulate Matter Air Pollution

**DOI:** 10.1371/journal.pone.0068829

**Published:** 2013-07-10

**Authors:** Holly H. Chiu, Peter Whittaker

**Affiliations:** 1 Department of Pharmacy, Harper University Hospital, Detroit Medical Center, Detroit, Michigan, United States of America; 2 Cardiovascular Research Institute and Department of Emergency Medicine, Wayne State University, Detroit, Michigan, United States of America; Maastricht University Medical Center, The Netherlands

## Abstract

**Background:**

Emerging evidence, mainly from Europe and Asia, indicates that venous thromboembolism (VTE) occurs most often in winter. Factors implicated in such seasonality are low temperature-mediated exacerbation of coagulation and high levels of particulate matter (PM) air pollution. However, in contrast to most European and Asian cities, particulate matter pollution peaks in the summer in many North American cities.

**Objectives:**

We aimed to exploit this geographical difference and examine the temporal distribution of VTE in a cold-weather, North American city, Detroit, with a summer PM peak. Our goal was thereby to resolve the influence of temperature and PM levels on VTE.

**Methods:**

Our retrospective, analytical semi-ecological study used chart review to confirm 1,907 acute, ambulatory VTE cases, divided them by location (Detroit versus suburban), and plotted monthly VTE frequency distributions. We used Environmental Protection Agency data to determine the temporal distribution of PM pollution components in Detroit. Suburban PM air pollution is presumed negligible and therefore not monitored.

**Results:**

Acute VTE cases in Detroit (1,490) exhibited a summer peak (June 24^th^) and differed from both a uniform distribution (*P*<0.01) and also that of 1,123 no-VTE cases (*P*<0.02). Levels of 10 µm diameter PM and coarse particle (2.5 to 10 µm) PM also exhibited summer peaks versus a winter peak for 2.5 µm diameter PM. Contrary to their urban counterparts, suburban cases of acute VTE (417) showed no monthly variation.

**Conclusions:**

The summer peak of acute VTE in Detroit indicates that low temperature is not a major factor in VTE pathogenesis. In contrast, concordance of the 10 µm diameter PM, coarse particle, and the Detroit VTE monthly distributions, combined with no monthly suburban VTE variation, is consistent with a role for PM pollution. Furthermore, divergence of the VTE and 2.5 µm PM distributions suggests that particle size may play a role.

## Introduction

Acute and long-term exposure to air pollution can exert adverse effects on cardiovascular health [1]. Although air pollution takes many forms, its cardiovascular effects have generally been attributed to particulate matter (PM) and, specifically, different sizes of PM. Because such particles are usually irregularly shaped, they are characterized by aerodynamic diameter rather than geometric diameter and are divided into three categories; fine particles with diameter less than 2.5 µm (PM_2.5_), those with diameter less than 10 µm (PM_10_), and coarse particles (PM_10_– PM_2.5_). PM exposure, particularly PM_2.5_, has been associated with increased risk of myocardial infarction [2] and hospital admissions for heart failure [3]. There is also evidence that PM pollution provokes venous thromboembolism (VTE) [4].

A recent meta-analysis concluded that the occurrence of VTE exhibited seasonal variation with a winter peak [5]; a suggestive finding because parallel seasonal variations in PM occurred in the cities where the studies were conducted. Nevertheless, other potential influences such as cold weather-induced coagulation enhancement cannot be discounted. Seasonal variation in PM differs in different locations [6] and therefore it may be possible to resolve the influences of the proposed pollution and temperature mechanisms. Our hypothesis was that Detroit provides an opportune location to differentiate these mechanisms because it is a city with relatively high PM levels that peak in the summer [7], [8]. Therefore, we aimed to determine if the occurrence of VTE in Detroit exhibited seasonal variation, and if variation in VTE corresponded to PM variation.

## Methods

Our study was approved by the Wayne State University Human Investigation Committee and the Detroit Medical Center. Because the study was a retrospective one, we requested and were granted a waiver to not obtain written consent from the patients. We used an analytical semi-ecological study approach [9]. This is a standard format in many air pollution studies; one in which the outcomes are measured in individuals, while the exposure is assessed at the group level.

We used the International Classification of Diseases, 9^th^ revision Clinical Modification (ICD-9-CM) codes (415.x, 451.x and 453.x) to identify potential VTE cases that presented to the emergency department at Detroit Medical Center hospitals from 2004 to 2008. Three hospitals (Detroit Receiving, Harper University, and Sinai-Grace) are located within the city of Detroit (Wayne County), while one (Huron Valley-Sinai) is located in a suburban area (Commerce Township, Oakland County) at the northwestern edge of the Detroit Metropolitan region, approximately 40 km northwest of the other hospitals.

From the cases identified, we excluded patients transferred from nursing homes and other hospitals, patients who had in-hospital VTE, and patients younger than 18 years of age. We included only ambulatory cases of acute VTE to maximize the PM exposure of the population and to minimize the influence of the VTE risk factor of immobility.

From the remaining VTE events, we examined the electronic medical record (specifically the radiology report) to determine if acute VTE was present; deep vein thrombosis (DVT) was confirmed by sonography and pulmonary embolus (PE) was confirmed by either CT scan or, in a small number of cases (n = 24), a ventilation/perfusion lung scan using scintigraphy. We included cases of upper limb DVT, but excluded cases of pelvic vein and saphenous vein thrombosis. We also excluded cases of DVT that were judged by the radiologist to represent ‘old thrombosis’ – so-called chronic DVT; i.e., no acute thrombosis was identified or no change was deemed to have occurred versus a previously performed ultrasound examination. The diagnosis of chronic rather than acute DVT is typically made when certain features are present. For example, if one or more of the following is found; vein walls that are thickened and irregular in dimension, recanalization of the thrombus, increased echogenicity and calcification of the thrombus, and the presence of collateral vessels [10].

In addition to the chronic DVT and the pelvic and saphenous vein occlusion cases already mentioned, the rest of the exclusions (even though all cases had been assigned a VTE-related ICD-9-CM code) did not have a confirmed VTE. The most frequent discharge diagnoses in such cases were phlebitis and shortness-of-breath. All of these excluded cases, after division according to hospital location, formed our no-VTE comparator groups.

For the confirmed VTE cases, we recorded the following information from the electronic medical record: the date of hospital visit, gender, self-reported ethnicity, age, height, body mass, body mass index (BMI), smoking history, comorbidities, and if the patient had prior VTE, current malignancy, or surgery or trauma within 90 days preceding the hospital visit. Our assessment of the electronic medical records to obtain this information was not restricted to the emergency department notes, but extended to the history and physical, consultation, radiology, and discharge notes too. In addition, records from both prior and subsequent hospital visits as well as outpatient clinic visits were also examined. We abstracted the information onto a single-page data-collection form and subsequently coded the data to an Excel file (Microsoft Office 2007, Microsoft Corp., Redmond, WA). Because additional data was subsequently collected from the same cases for use in other studies, we also performed quality control checks for the recorded information on all of the cases.

For the no-VTE cases, we recorded the date of the hospital visit, gender, self-reported ethnicity, and age. If available, we also noted height, weight, and BMI; however, because these patients were often not admitted to the hospital, detailed histories were seldom taken and so we did not attempt to record any other information.

Cases from the three Detroit hospitals (data combined and subsequently referred to as “Detroit”) and from Huron Valley-Sinai Hospital (referred to as “suburban”) were divided into VTE and no-VTE groups.

### Temporal Distribution of VTE

We determined the number of VTE cases per calendar month and then calculated the monthly proportion expressed as a percentage of the total number of cases. The same procedure was done for the comparator (no-VTE) cases. Because the expectation, based on previous studies, was that the majority of cases would occur in the winter, we first calculated the odds ratio for cases in a specific month versus winter (the average number of cases that occurred in December, January, and February). We then determined if the monthly distributions of the VTE cases and the comparator groups were uniform and also compared the distributions. Finally, we determined if the monthly distributions correlated with a periodic regression.

### Relationship between Symptom Onset and Hospital Arrival

We recorded the time between when patients first noted symptoms (as stated in the electronic medical record notes) and their hospital arrival. We divided the delays as follows: less than 1 day, 1–2 days, 3–7 days, 1–2 weeks, more than 2 weeks, and not reported.

### Air Pollution and VTE

To determine if there was an association between monthly VTE distribution and the PM parameters, we examined data from the United States Environmental Protection Agency (EPA) website (http://www.epa.gov/airdata/). There are several air-quality monitoring stations located within the Detroit metropolitan area; however, not all stations measure all PM parameters. We therefore analyzed data from the station closest to the Detroit hospitals from which all PM parameters were available (located in the city of Dearborn: site identification 26-163-0033; 8–12 km from the hospitals). The closest monitoring station to the suburban hospital was >25 km away and did not record PM_10_ data. Therefore, we could not directly assess the influence of PM on suburban VTE. Our assumption was that PM pollution at the suburban location was much less than in Detroit. For the urban site, we calculated the average weekly values of PM_10_, PM_2.5_, and coarse particles per year and then determined the average weekly value for the entire study period. We determined if these weekly distributions correlated with a periodic regression and compared the distribution of each PM parameter to the weekly VTE distribution.

### Statistical Analysis

The case characteristic data are presented as either means (for the continuous variables) or proportions (for the categorical variables) together with their respective 95% confidence intervals (CI).

We used circular statistics to analyze the temporal distribution of emergency department visits and PM parameters [11], [12]. These statistical methods are specifically designed to analyze data that possess spatial or temporal direction. We have previously used such methods to examine spatial effects; cardiac muscle disarray in hypertrophic cardiomyopathy [13] and changes in collagen fiber orientation produced by application of tension in tendons [14]. The same techniques can also be applied to the analysis of data with temporal distribution.

For VTE and no-VTE cases, we tested to determine if the monthly distributions were uniform. However, there are many uniformity tests and, because we had no reason to anticipate specific deviations from uniform (e.g., unimodal versus bimodal), we used two tests. First, we applied Kuiper’s modification of the Kolmogorov-Smirnov test; an omnibus test capable of identifying any deviation from uniformity [12]. Nevertheless, this test may be ineffective in detecting specific deviations from uniformity [12]. Some previous studies reported unimodal distribution of VTE monthly incidence and so we also applied the Rayleigh test; used to evaluate uniformity against a unimodal alternative. In addition, we determined if the temporal distributions possessed cyclical correlations; so-called periodic regression. To accomplish this, the dates were first converted to radians. We then calculated sine and cosine transforms to enable multiple regression to be performed. The coefficients thereby obtained were then used to construct the periodic regression curve [11]. An example of the calculations is provided in the supplemental material [Text S1]. Chi-squared goodness of fit tests were used to assess whether the observed temporal distributions were influenced by specific patient characteristics [15]. Kuiper’s two sample test was used to compare distributions [16].

Statistical analysis was conducted using Stata (Version 12.1, Statacorp, College Station, TX); however, we used algorithms created in Excel (Microsoft Office 2007, Microsoft Corp., Redmond, WA) to perform the circular statistics component of the analysis.

## Results

### Study Population

**Figure 1:**
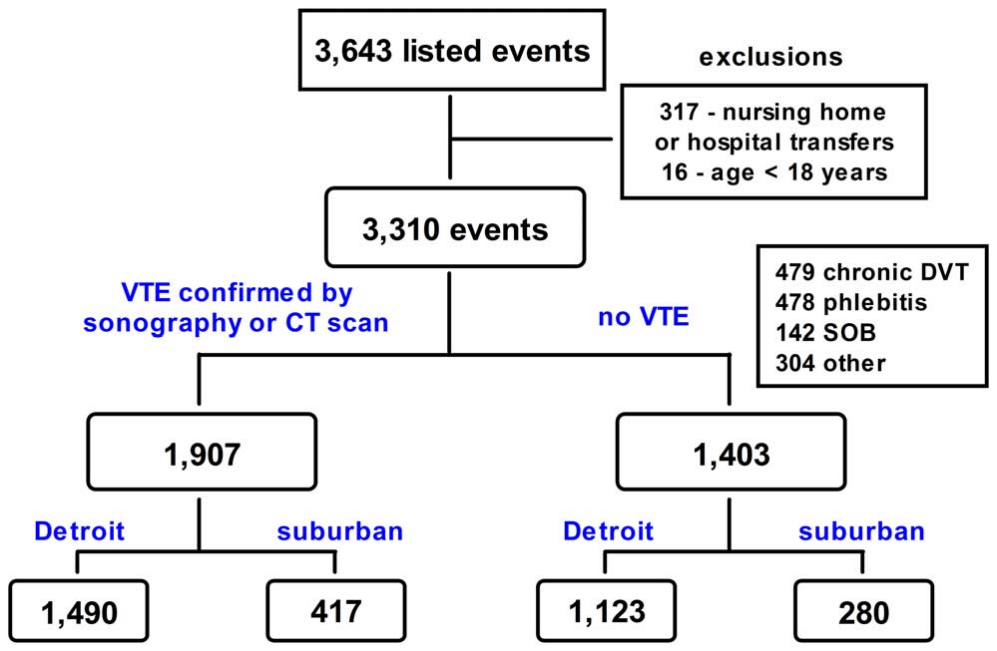
Case inclusion flow-chart. CT – computed tomography; DVT – deep vein thrombosis; SOB – shortness-of-breath; VTE – venous thromboembolism.

We identified 3,643 events (Figure 1). After exclusion for inter-hospital and nursing home transfers and age, 3,310 events from 3,091 patients remained; i.e., some patients made multiple emergency department visits. Most of these 219 repeat visits were not for acute VTE, but were usually diagnosed as chronic DVT and most of these were made by a small group; 25 individuals each made ≥5 visits. Each separate event, whether it was determined to be a case of VTE or not, was included in the analysis of the appropriate group. The majority of the no-VTE cases were judged to be chronic DVT or phlebitis (Figure 1). The main components of the ‘other’ category were; chest pain/rule out PE (n = 86), leg pain/rule out DVT (n = 79), cellulitis (n = 44), prescription refill (n = 26), and check of international normalized ratio (n = 18).

The odds ratio for confirmed VTE in Detroit versus the suburban location was 0.89 (95% CI 0.75–1.06); indicative of no major location-associated difference in the ICD-9-CM coding and identification process.

### Case Characteristics

Complete characteristic data were available for ∼80% of the acute VTE cases. In contrast, characteristic data were available for only ∼30% of the no-VTE cases (Table 1); as anticipated in a population seldom admitted to hospital. In both groups, the missing data were for height, body mass, and hence also BMI.

**Table 1:** Case characteristics.

	Detroit VTE	Detroit no-VTE	Suburban VTE	Suburban no-VTE
	[n = 1,490]	[n = 1,123]	[n = 417]	[n = 280]
**Female**	52 (49, 54)	49 (46, 52)	55 (50, 60)	69 (63, 74)
**African ancestry**	84 (82, 86)	85 (83, 87)	5 (3, 7)	2 (0, 4)
**Age (years)**	54 (53, 55)	50 (49, 51)	60 (58, 62)	50 (48, 52)
**Height (m)**	1.71 (1.71, 1.72)	1.72 (1.71, 173)	1.70 (1.69, 1.72)	1.71 (1.68, 1.74)
	[1,229]	[384]	[341]	[58]
**Body mass (kg)**	87 (85, 88)	87 (85, 89)	86 (84, 89)	87 (81, 94)
	[1,266]	[381]	[361]	[60]
**BMI (kg/m^2^)**	29.7 (29.2, 30.1)	29.3 (28.5, 30.1)	29.6 (28.8, 30.5)	29.9 (27.9, 32.0)
	[1,226]	[370]	[338]	[58]
**Smoking history**	47 (44, 49)	–	24 (19, 28)	–
**Diabetes mellitus**	18 (16, 20)	–	11 (8, 14)	–
**DVT/PE/both (n)**	956/403/131	–	274/93/50	–
**Previous VTE**	33 (30, 35)	–	25 (21, 30)	–
**Malignancy**	20 (18, 22)	–	22 (18, 26)	–
**Surgery/trauma**	14 (12, 15)	–	21 (17, 25)	–
**Idiopathic**	68 (66, 70)	–	60 (58, 62)	–

Parameters without units are expressed as percentages with 95% confidence intervals in parentheses except for indication; for which the n-values are given. Parameters with units are shown as means with 95% confidence intervals in parentheses. Values in square brackets indicate sample size – if no sample size is given, there were no missing data and the sample size corresponds to the value at the top of the column. Idiopathic indicates a venous thromboembolism that occurred when the patient did not have malignancy and had not had surgery or trauma within the preceding 90 days. BMI, body mass index; VTE, venous thromboembolism; DVT, deep vein thrombosis; PE, pulmonary embolism.

For VTE cases, there were inter-site differences in ethnicity, age, history of smoking, and prevalence of diabetes mellitus; however, there were no differences in height, body mass, or BMI (Table 1). The Detroit population was predominantly of African ancestry, while the suburban population contained few such patients. Patients with VTE were older than the corresponding no-VTE groups. Detroit cases with VTE were younger than their suburban counterparts. Approximately two-thirds of the confirmed cases were DVT: Detroit 64% versus suburban 66%. Although there was no inter-site difference in the proportion of patients who had current malignancy, a greater proportion of suburban patients had recent surgery or trauma and therefore there was a corresponding higher proportion of idiopathic cases (defined as those without malignancy and without recent surgery or trauma) in Detroit. The proportion of cases with a prior VTE was higher in Detroit. The proportion of upper limb DVT cases was similar between sites; Detroit 5.7% (n = 85) and suburban 5.3% (n = 22). No-VTE cases differed in their reported discharge diagnosis (*P*<0.0001; chi-squared test); in Detroit, the three most frequent were chronic DVT (40%), thrombophlebitis (24%), and shortness-of-breath (12%) versus 9% chronic DVT, 76% thrombophlebitis, and 2% shortness-of-breath at the suburban location.

### Temporal Distribution of VTE

**Figure 2:**
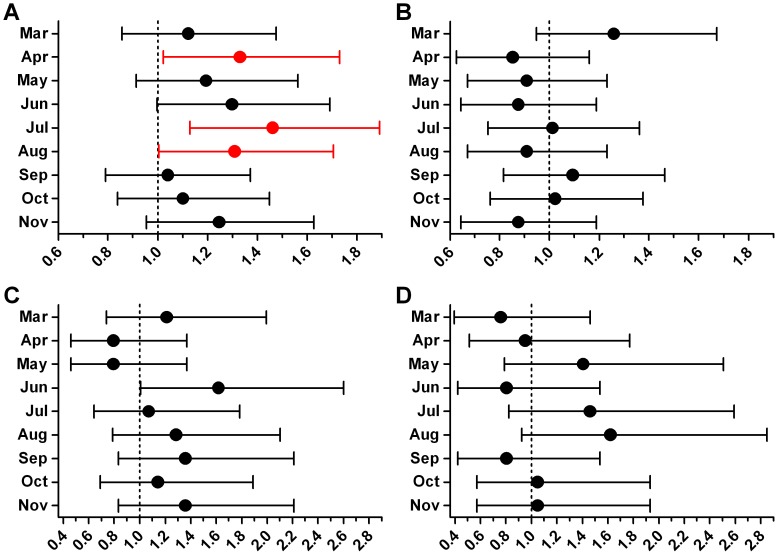
Odds ratio for each month versus winter. Data are shown with 95% confidence intervals and red denotes a statistically significant difference (*P*<0.05) versus winter (defined as the average number of cases in December, January, and February). (A) Detroit VTE. (B) Detroit no-VTE. (C) Suburban VTE. (D) Suburban no-VTE.

The odds ratios for the monthly Detroit VTE cases versus the winter average were all above 1.0 (Figure 2A); a finding inconsistent with a winter peak in VTE. In contrast, there was no discernible pattern for any of the other groups (Figure 2BCD). Additionally, the only statistically significant differences occurred in the Detroit VTE group.

**Figure 3:**
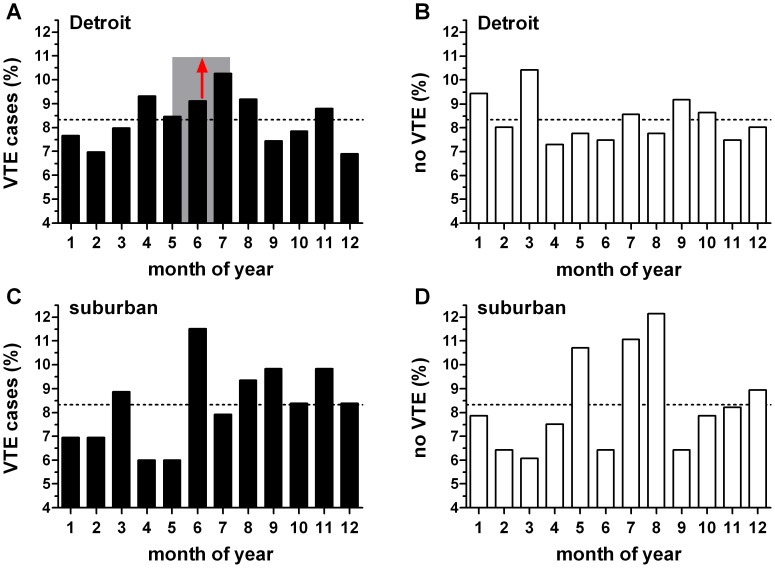
Distribution of monthly venous thromboembolism (VTE) and no-VTE cases. X-axis indicates the month of the year (January = 1). Dashed line indicates uniform distribution. (A) Detroit VTE (n = 1,490). This was the only distribution that differed from uniform (*P* = 0.0046; Rayleigh test). Distribution mean (red arrow) June 24^th^; the 95% confidence intervals (May 19^th^ to July 31^st^) are indicated by the shaded rectangle. (B) Distribution of Detroit cases that did not have a VTE (n = 1,123); did not differ from uniform (*P* = 0.50), but differed from the Detroit VTE cases (*P*<0.02; Kuiper’s test). (C) Suburban VTE (n = 417); did not differ from uniform (*P* = 0.13). (D) Suburban cases that did not have a VTE (n = 280); did not differ from uniform (*P* = 0.21) or the suburban VTE distribution.

We found the monthly distribution of VTE at the Detroit hospitals differed from uniform when assessed by both the Kuiper (*P*<0.05) and Rayleigh tests (*P* = 0.0046). The shape of the distribution indicated greater incidence of VTE in summer (distribution mean: June 24^th^, 95% CI May 19^th^, July 31^st^) than in winter (Figure 3A). Table 2 shows our analysis of the distribution of risk factors examined as a function of season. As anticipated from the previous result, the distribution of VTE cases differed from the 25% per season expected for a uniform distribution (*P* = 0.002; chi-squared goodness-of-fit test). For the listed risk factors, our expected (comparison) distribution was that of the observed VTE distribution. We found no significant differences between the observed and expected distributions for any of these parameters. Similarly, there was no seasonal difference in the distribution of case age or BMI.

**Table 2:** Seasonal distribution of venous thromboembolism in Detroit as a function of potential risk factors.

	Winter	Spring	Summer	Autumn	?^2^	*P*-value
**VTE (1,490)**	323 (21.7)	384 (25.8)	424 (28.5)	359 (24.1)	14.52	0.002
**African ancestry (1,250)**	276 (22.1)	322 (25.8)	354 (28.3)	298 (23.8)	0.14	0.99
**Women (767)**	155 (20.2)	199 (25.9)	218 (28.4)	195 (25.4)	1.34	0.72
**Smoking history (694)**	161 (23.2)	174 (25.1)	192 (27.7)	167 (24.1)	1.03	0.79
**Hypertension (779)**	170 (21.8)	203 (26.1)	216 (27.7)	190 (24.4)	0.21	0.98
**Diabetes mellitus (263)**	54 (20.5)	70 (26.6)	85 (32.3)	54 (20.5)	3.00	0.39
**Obesity (503)**	105 (20.9)	131 (26.0)	140 (27.8)	127 (25.2)	0.51	0.92
**Surgery/trauma (201)**	32 (15.9)	57 (28.4)	66 (32.8)	46 (22.9)	5.08	0.17
**Malignancy (295)**	56 (19.0)	70 (23.7)	93 (31.5)	76 (25.8)	2.78	0.43
**Age (1,490)**	52 (42, 63)	52 (41,67)	52 (41,67)	54 (42, 66)	–	0.58
**BMI (1,226)**	27.9 (24.0, 33.9)	28.2 (23.8, 34.0)	28.1 (23.7,33.9)	28.3 (24.5, 33.9)	–	0.95

For the venous thromboembolism (VTE) cases, the expected distribution against which the observed frequencies were tested was 25% per season. For the categorical potential risk factors, the expected distribution was that of the observed VTE cases. Categorical parameters are expressed as absolute values followed by the percentage in parentheses. Continuous variables are shown as medians followed by interquartile ranges in parentheses. The sample size for each category is shown in parentheses after the category label. Winter – December, January, February; spring – March, April, May; summer – June, July, August; autumn – September, October, November. Obesity was defined as a body mass index (BMI) >30 kg/m^2^.

In contrast to the Detroit acute VTE cases, the distribution of no-VTE cases did not differ from uniform (Figure 3B). Furthermore, comparison of the Detroit VTE and no-VTE distributions revealed a difference (*P*<0.02; Kuiper’s test). In contrast, the distribution of suburban VTE cases and the suburban cases of no-VTE did not differ from uniform (Figures 3C and 3D) and there was no difference between these two distributions.

**Figure 4:**
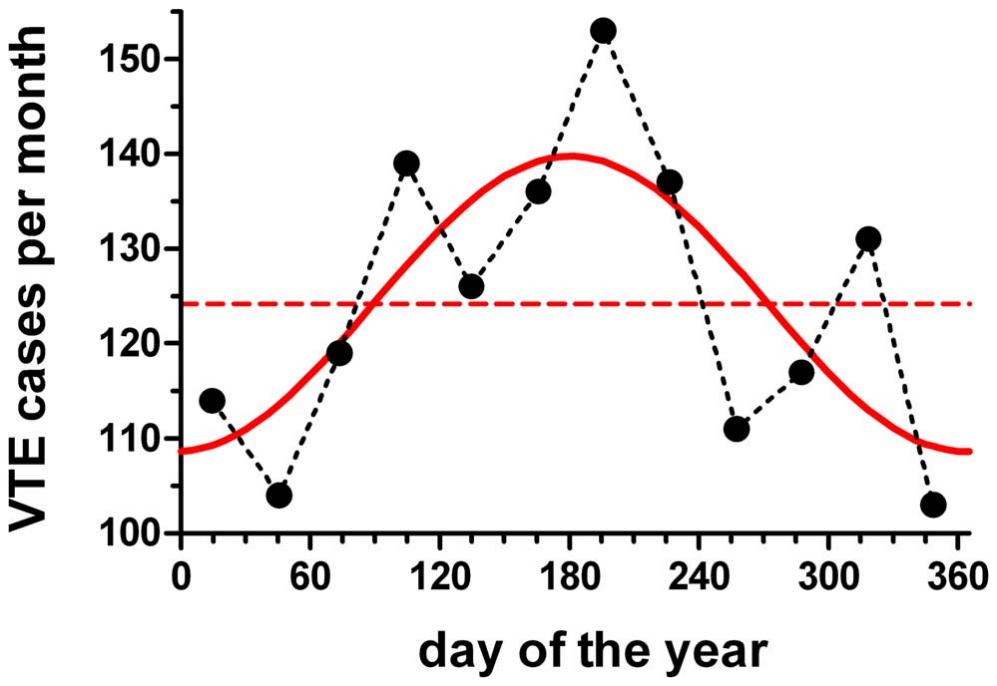
Periodic regression of venous thromboembolism (VTE) cases (Detroit). Black circles indicate the monthly averages. The periodic regression is shown in red (*P* = 0.028; R^2^ = 0.55); June 30^th^ peak. Periodic mean = 124.2 cases/month (dashed red line).

The monthly VTE distribution in Detroit also exhibited a correlation with a sine wave (*P* = 0.028, R^2^ = 0.55; periodic regression analysis; Figure 4). The peak of the fitted function occurred on June 30^th^. In contrast, no correlations were found for the other groups: Detroit no-VTE cases *P* = 0.71; suburban VTE *P* = 0.19; suburban no-VTE cases *P* = 0.30 (periodic regression analysis).

### Relationship between Symptom Onset and Hospital Arrival

**Figure 5:**
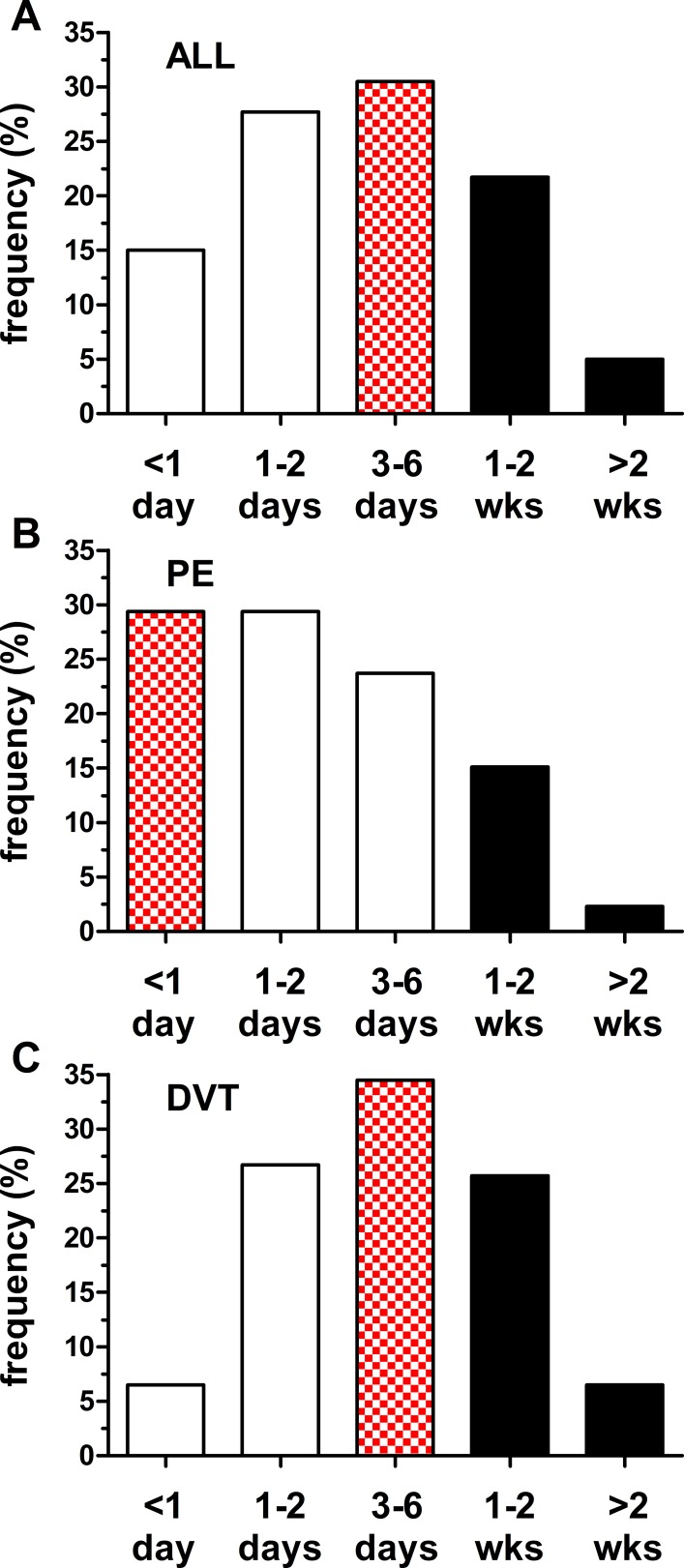
Delay from self-reported symptom onset to emergency department arrival. (A) All cases with recorded times (n = 1,504). The mode (chequered bar; 31%) occurred at 3–6 days; however, the delay exceeded one week for more than 25% of cases (black bars). (B) Pulmonary embolus (n = 557). The mode (29%) occurred in the <1 day period; proportion with delays exceeding one week was 17%. (C) Deep vein thrombosis (n = 947). The mode (35%) occurred at 3–6 days; proportion with delays exceeding one week was 32%. The distribution for deep vein thrombosis differed from that for pulmonary embolus (*P*<0.001; Kuiper’s two sample test).

The delay between self-reported onset of symptoms and hospital arrival was recorded for most patients (n = 1,504; 79%). For all VTE cases, the mode (31%) was 3–6 days; nevertheless, the delay exceeded one week for a large proportion (27%; Figure 5A). The delay was shorter for PE (n = 557) than DVT (n = 947) cases: the majority of patients with PE (59%; Figure 5B) had a delay of less than 3 days versus 33% of patients with DVT (Figure 5C). The delay distributions for PE and DVT differed (*P*<0.001; Kuiper’s test); however, we found no difference between the delay distributions for Detroit and the suburban location (data not shown).

### Potential Air Pollution Determinants of VTE Distribution

**Figure 6:**
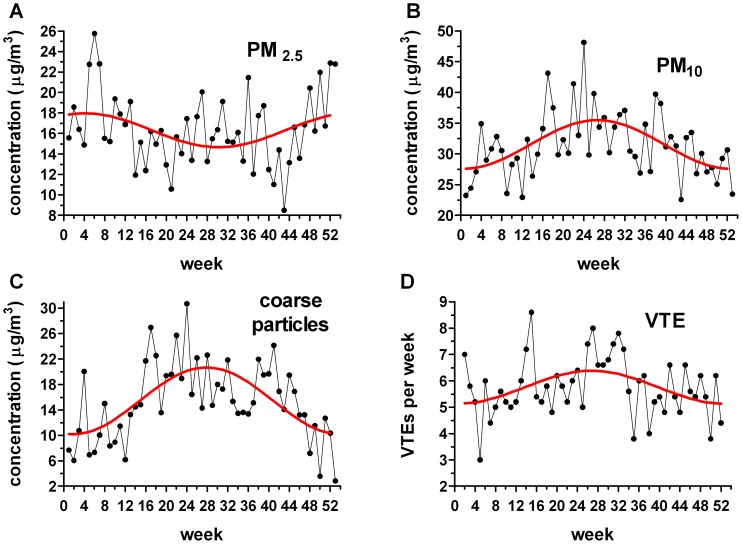
Weekly distribution of particulate matter (PM) parameters and venous thromboembolism (VTE) cases (Detroit hospitals). Periodic regressions are shown in red. (A) 2.5 µm PM (*P* = 0.049; R^2^ = 0.12); January 26^th^ peak. (B) 10 µm PM (*P* = 0.0003; R^2^ = 0.28); July 2^nd^ peak. (C) Coarse particles (*P*<0.0001; R^2^ = 0.40); July 11^th^ peak. (D) VTE cases per week per year (*P* = 0.018; R^2^ = 0.15); July 3^rd^ peak.

On the basis of the delay results and to enable incorporation of the variability of the delay into the analysis, we assessed PM data when calculated weekly rather than monthly. The weekly PM distributions exhibited different periodicities (Figure 6). PM_2.5_ possessed a relatively weak periodic correlation (*P* = 0.049; R^2^ = 0.12; periodic regression analysis) with a winter peak (January 26^th^). In contrast, while PM_10_ and coarse particles also correlated with periodic functions (*P* = 0.0003; R^2^ = 0.28 and *P*<0.0001; R^2^ = 0.40; periodic regression analysis), both had summer peaks (July 2^nd^ and 11^th^ respectively). As expected, the weekly VTE distribution also possessed a periodic regression (*P* = 0.018; R^2^ = 0.15; peak July 3^rd^); consistent with the monthly distribution described previously.

We compared the cumulative frequency distribution of the weekly VTE data with the cumulative frequency distribution of each PM parameter using Kuiper’s two-sample test. In this analysis, a difference between PM and VTE distributions would suggest no association. For both coarse particles and PM_2.5_, the difference versus the VTE distribution was large (*P*<0.001); indicative of no temporal association between VTE and these PM components. For PM_2.5_, we attribute the difference to its winter peak versus the VTE summer peak. Even though course particles, like VTE, exhibited a summer peak, the statistical difference was attributed to the greater amplitude of the coarse particle’s periodic regression (Figure 6C). A lateral shift of the VTE distribution to accommodate delay between symptom onset and diagnosis would not improve the correspondence of these curves. In contrast to the other PM parameters, there was no difference between the PM_10_ and VTE distributions (*P*>0.50; Kuiper’s two-sample test); indicative of a temporal association.

## Discussion

We found significant periodic variation in VTE occurrence during the year with a summer peak for cases treated at hospitals located within Detroit. In contrast, we found no periodic variation in VTE occurrence at a suburban hospital at the edge of the Detroit metropolitan region. Periodic variation was also found in PM pollution monitored at a site close to the Detroit hospitals; PM_10_ and coarse particles exhibited summer peaks, while PM_2.5_ had a winter peak.

Our results are in stark contrast to most previous reports; but, as such, we propose that they provide insight into potential relationships between VTE and PM pollution. A meta-analysis of 17 studies that assessed seasonal or monthly distribution of VTE and included more than 25,000 patients consistently found increased incidence of VTE in the winter and the lowest number of cases in July [5]. How can such discordant results be reconciled?

First, it should be noted that 12 studies in the meta-analysis were European and the remaining five were Asian; China (2), Turkey (2), and Iran. Because seasonal variation in PM differs between locations, our results may not be discordant. The majority of cases in the meta-analysis came from the Emilia Romagna region of northern Italy [17]; documented to have winter PM peaks [18]. Higher winter levels of PM are a relatively consistent finding in European cities and are attributed to air temperature inversions that trap pollution close to the ground. Such inversions occur most often in winter; summer is usually associated with vertical mixing of the air. For example, six of seven Swiss sites (including urban, suburban, and rural) had higher winter levels of PM_10_ and PM_2.5_ versus summer [19]. The exception was a location 1,140 m above sea level (i.e., above the winter inversion layer) that had a summer peak. Similarly, the Asian cities included in the meta-analysis all have higher winter PM levels [20], [21], [22], [23], [24]. In contrast, PM_10_ levels in the United States tend to be highest in summer, albeit with regional and local variation; for 1987–2000, summer peaks were found in the Northeast, Industrial Midwest, Southeast, and Upper Midwest and autumn peaks in the Northwest, Southwest, and Southern California [6]. Therefore, if PM plays a crucial role in VTE pathogenesis, our results are not discordant with previous European and Asian findings.

In fact, rather than being a source of confusion, such regional differences in PM and VTE variation may enable evaluation of the role of environmental factors through a process of elimination. For example, several studies that reported winter peaks in VTE attributed a potential role to low temperature-mediated enhancement of coagulation [17]. Our finding of a summer VTE peak appears to discount this possibility. Variation in another meteorological parameter, atmospheric pressure, has also been implicated. A Scottish study of 37,336 cases found an association between higher winter incidence of DVT and lower pressure [25]. The average seasonal pressure in Detroit over the course of our study was lower in summer than winter (1,015±1 hPa versus 1,018±3 hPa; *P*<0.05 by ANOVA) and so there was a similar association between lower pressure and increased incidence of VTE. Nevertheless, atmospheric pressure appears an unlikely influence because our suburban population was exposed to the same pressure variation without resultant variation in VTE. In addition, although the summer versus winter pressure difference was statistically significant, it was small and the intra-month variation was typically greater. The observed difference in the monthly VTE distributions between the urban and suburban locations may eliminate all meteorological parameters as responsible factors because exposure would have been almost identical in both locations.

We attempted to address potential bias from another possible source of temporal variation (overall monthly emergency department visits) by inclusion of comparator groups. Comparator groups have seldom been employed in this type of study; probably because case selection is problematic. There are many conditions that exhibit seasonal variation (for example, influenza) and also other conditions that may be triggered by seasonal increases in levels of PM pollution (for example, asthma); thereby making comparator selection susceptible to bias. In the United States, emergency department visits occur more frequently in summer; for example, in 2008, 26.8% (June 21^st^ to September 22^nd^) versus 22.9% in winter (December 22^nd^ to March 19^th^); from http://www.cdc.gov/nchs/data/ahcd/nhamcs_emergency/2008_ed_web_tables.pdf. Thus, a summer peak in VTE may be reflection of this pattern. However, a summer peak would then also be expected for the ICD-9-code-identified cases that were found not to have a VTE; that is, the comparator groups’ monthly distribution would parallel that of the VTE cases. This was not the case and the monthly distributions of the no-VTE comparator and VTE groups in Detroit were significantly different. Therefore, we do not believe that our results were confounded by temporal variations in overall emergency department visits.

At first sight, our results appear incompatible with PM_2.5_ playing a role because of its winter peak. However, we cannot rule out the possible influence of PM_2.5_ completely for two reasons. First, the composition of PM_2.5_ is known to vary. For example, the Dearborn monitoring site assesses speciation of PM_2.5_; data from 2004 revealed a multitude of elements with ammonium, elemental carbon, iron, organic carbon, sulphate, sulphur, and nitrate the chief constituents. Components of PM_2.5_ also vary by season and region [26]. Second, the recorded PM_10_ levels include PM_2.5_ also and so without direct measurement of coarse particles (in our study derived simply as the subtraction of PM_2.5_ from PM_10_), there is potential for misinterpretation. Such complexity illustrates the danger of simplistic interpretation of associations (or their absence) between PM data and health-related outcomes.

It is possible that attribution of meteorological and PM-related influences may be attenuated or amplified by economic factors. The average household income for the zip-codes of the Detroit hospitals was $12,262 and $40,041 versus $78,982 for the suburban location (2010 census data). Income disparity may produce differences in home heating and cooling; for example, cardiovascular-related hospital admissions associated with high PM_2.5_ levels were reduced in communities with a higher prevalence of air conditioning [27]. In addition, neighborhood deprivation has been identified as a risk factor for VTE [28] and so it is possible that, given the large apparent disparity in income, some as-yet unidentified income-associated factor may serve as a seasonal trigger.

Despite our finding of an apparent lack of seasonal influence by potential VTE risk factors (Table 2), two additional factors argue against a causal link between PM pollution and VTE. First, over the course of our study, there was, albeit with inter-year fluctuation, an overall decrease in PM in Detroit. Nevertheless, the number of VTE cases increased. This divergence appears contradictory; however, there are potential explanations. It is possible that VTE is triggered by a threshold value of PM (either via single exposure or a sustained period of increased levels) and so, even though the average declined, it may still be above the required threshold. Similarly, peaks in daily values above such a threshold may have still occurred (even though the average daily value declined). Our observed increase in VTE cases may also reflect the overall reported increase in VTE incidence [29]. The increase in VTE may mirror changes in socioeconomic factors too; for example, an increase in emergency department use for primary care. Finally, changes in coding practices may have contributed to the increase. The second issue is that although Detroit has high levels of PM relative to the rest of the country, there are many cities in the world with higher levels. For example, average PM_10_ values for the ‘dry season’ (∼November-April) were 262 µg/m^3^ in Beijing and 186 µg/m^3^ in Hanoi [20]; much higher than even the peak weekly average of ∼50 µg/m^3^ in Detroit (Figure 6B). Nevertheless, we are unaware of studies that indicate an epidemic of VTE in these or other cities with similarly high PM pollution. This information could indicate that there is little or no connection between VTE and PM. Conversely, the information could indicate that PM is a factor that only provokes VTE in vulnerable populations; a concept that has garnered recent attention for other health effects [30]. Therefore, if the heavily polluted cities are inhabited by a younger, slimmer, and less sedentary populace with healthier diets and lacking some of the genetic [31] or other risk factors associated with the population of Western cities where pollution has been implicated in VTE, this might explain the discrepancy.

Because resolution of this apparent inconsistency is fundamental to the role of PM in VTE, we performed additional post-hoc analysis. We hypothesized that if PM is a trigger in potentially vulnerable populations, we would see more pronounced seasonal variation in such groups. We examined three VTE risk parameters available from our data; age, BMI, and the presence of non-idiopathic VTE (cases with current malignancy, recent surgery, or recent trauma). For the former two groups, we analyzed cases in patients older than 70 years and cases with BMI ≥34kg/m^2^; values selected to maintain a reasonably large sample. The distribution for older cases (n = 304) differed from uniform (*P* = 0.026; Rayleigh test), but obese cases (n = 305) did not (*P* = 0.15). However, even the significant result for the older cases did not represent a more pronounced seasonal variation than seen in the whole population and so neither risk characteristic seemed associated with enhanced vulnerability.

**Figure 7:**
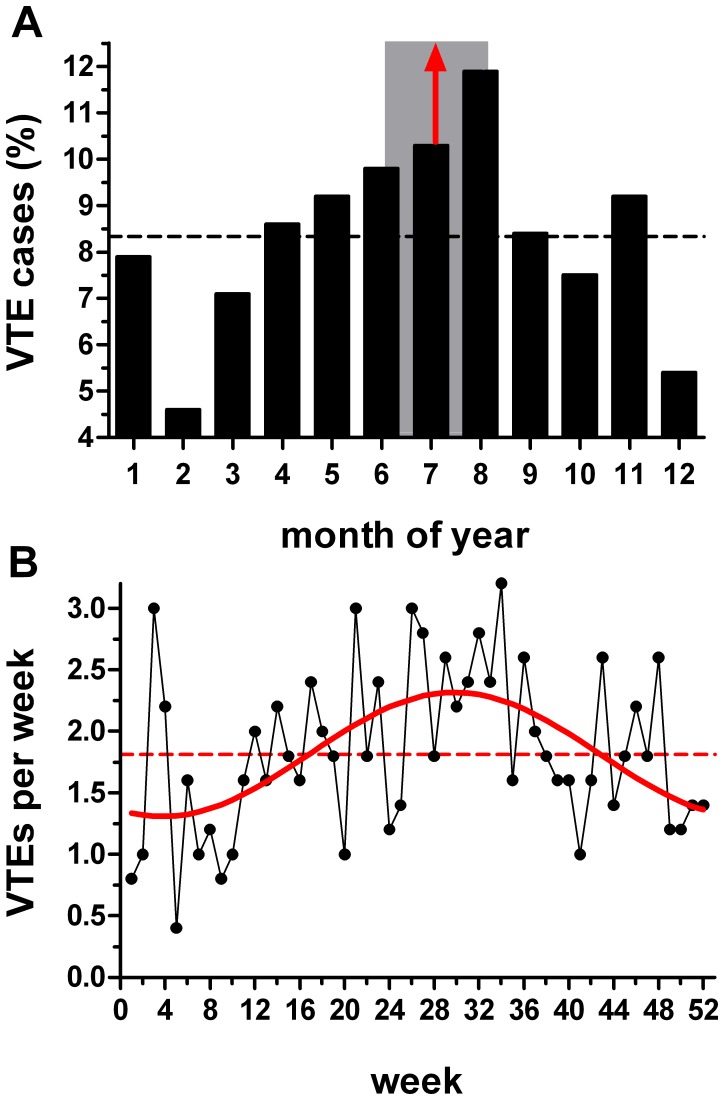
Distribution of non-idiopathic venous thromboembolism (VTE). (A) Monthly distribution (January = 1): dashed line indicates uniform distribution. Distribution differed from uniform (*P*<0.05 Kuiper’s test; *P* = 0.0004 Rayleigh test); mean (red arrow) = July 21^st^ (shaded rectangle indicates the 95% confidence intervals; June 21^st^ to August 20^th^). (B) Weekly distribution. Solid red line shows the periodic regression (*P* = 0.0004; R^2^ = 0.27); July 25^th^ peak.

In contrast, the non-idiopathic group (n = 478) had a monthly VTE distribution that differed from uniform (*P* = 0.0004; Rayleigh test; peak July 21^st^; 95% CI - June 21^st^, August 20th; Figure 7A), while the weekly distribution possessed periodic regression (*P* = 0.0004; R^2^ = 0.27; Figure 7B). These distributions exhibited more pronounced seasonal variation than observed in the entire population (compare with Figures 3A and 6D). There is evidence that the rate of malignancy and surgery in Asia is lower than in the United States and Europe. For example, the estimated 5-year prevalence of cancer in 2008 was higher in North America (1.70%) and in Western, Northern, and Southern Europe (1.49–1.90%) than in the Asian regions where the cities with high PM pollution are located (0.23–0.56%) [32]. There is a similar difference in the estimated surgical rate between these regions [33]. The apparent increased susceptibility of non-idiopathic cases to summer VTE merits further evaluation and may resolve the “Asian incongruity”.

### Spatial and Temporal Considerations

Attempts to attribute environmental causes to VTE must address two additional issues: the spatial and temporal relationships between the proposed trigger(s) and the exposed population. The challenges posed by the assumptions made to accommodate these factors make attribution of cause and effect difficult and may explain why some studies failed to find periodicity. Spatially, the assumption made is that the trigger is homogeneously distributed. This assumption is reasonable for factors such as ambient temperature; if the area is not too large. In contrast, the assumption of air pollution homogeneity is often less reasonable. Our data indicate a difference in the monthly distribution of VTE at locations within the same relatively small region; a difference possibly caused by non-homogeneous PM distribution.

Some of the inconsistency in reports of variability in VTE occurrence may be explained by such assumptions. For example, Stein et al. examined more than 7.5 million cases of VTE over 21 years obtained from the National Hospital Discharge Survey [34]. They found no seasonal (according to calendar quarters) variation in either DVT or PE for the entire United States or when the four regions of the country (West, Midwest, South, and Northeast; defined by the National Hospital Discharge Survey) were evaluated separately. The potential advantage of the large sample may have been negated by the large heterogeneity (both spatial and temporal) in environmental conditions, even within the same geographic region, and also by the inclusion of in-hospital VTE cases. Similarly, analysis of 26,450 patients in the Women’s Health Initiative Hormone Therapy trials also failed to find an association between PM exposure and VTE [35]. However, the number of VTE cases was relatively small (n = 508), average PM exposure was relatively low (PM_2.5_ ∼13.5 µg/m^3^; PM_10_ ∼27.0 µg/m^3^), and the study included patients from 40 centers throughout the United States. Again, we propose that environmental heterogeneity compromised detection of PM-mediated effects.

Another factor to consider is the delay between symptom onset and VTE diagnosis. Such delay, and especially its variability (0 to 14+ days in our study), will undermine attempts to correlate daily changes in PM (or any factor) to VTE occurrence unless the delay is incorporated into the analysis. We attempted to minimize the effect of variable delay by grouping VTE cases per week (the mode of the delay distribution). Nevertheless, a study of VTE (302 cases over two years) in Verona, Italy found a correlation between daily coarse particle levels and the daily number of VTE cases [36]. Incorporation of lag-times into this analysis failed to show a correlation with VTE. It is unclear why such correlation was found with no apparent delay. However, we speculate that because the authors removed the sequential temporal component from their analysis (that is, they plotted the number of VTE cases as a function of PM levels on specific dates), the general association of VTE occurrence with high PM levels could be sufficient to achieve statistical significance. For example, we too found a similar statistically significant linear correlation between weekly VTE cases and the weekly average of coarse particle level (*P* = 0.014; R^2^ = 0.11; data not shown), when plotted in the same manner as the Verona study. This correlation occurred even though we found no association between VTE incidence and coarse particle levels in the analysis of the cumulative frequency distributions; that is, when the data were plotted as a function of time. We propose that it is potentially misleading to remove the sequential, temporal component from analysis.

This example illustrates that data analysis is an issue in attempts to discern variation in VTE occurrence and correlation to environmental factors. Analysis methods have ranged from the simplistic to two-stage Bayesian models to correct for spatial misalignment in pollution data and subsequent bias [37]; but, no consensus has emerged regarding optimal approaches and few studies have used the same methodology.

### Clinical Relevance

Periodicity in VTE occurrence has potential implications. First, it raises the possibility that more than 12% of the summer VTE cases (Figure 4; (amplitude/periodic mean)×100%) might be avoidable if the trigger is identified and remedied. However, we cannot be sure if the increased summer incidence represents an absolute increase or a shift of cases from other months; that is, would patients have eventually had a VTE without exposure to the purported summer trigger? Our post-hoc analysis that implicated increased summer susceptibility in non-idiopathic cases (Figure 7) is compatible with the concept that absolute reductions in VTE could be achieved if vulnerable patients are protected. Second, periodicity may warrant increased suspicion for acute VTE in ambulatory patients whose symptoms are inconclusive at certain times of the year, depending upon local conditions; i.e., summer in Detroit. Third, our data together with the prior studies suggest that patients at risk for VTE might be advised not to go outside on days when PM pollution levels are high.

Efforts to assess the effect of air pollution on cardiovascular health have focused on PM_2.5_ and there is indeed a large amount of data to support this focus [1]. In fact, many EPA monitoring sites no longer measure PM_10_. Although all types of PM can activate inflammation, produce oxidative stress, and promote thrombosis and coagulation [1], [38], something specific to larger particles may be important in VTE as suggested by Martinelli et al. [36]. Although our results could be interpreted as support for a significant role of larger particles, we cannot rule out the possibility that some specific component of PM_2.5_ is important (even though the overall PM_2.5_ peak occurred in the winter).

### Limitations

We presented data from only one monitoring station and so it is possible that this location does not reflect pollution throughout the area. However, other stations in the catchment area of the Detroit hospitals reported similar seasonal variation (data not shown) and the summer peak of PM_10_ in Detroit has been noted by others [6], [8]. We have also made the assumption that the reported PM levels reflect individual exposure. Second, we have no direct PM data for the suburban location. We assumed that suburban PM levels were much lower than in Detroit because the location is less densely populated, has fewer major roads, and lacks the pollution sources that affect air quality in Detroit. The closest Oakland County monitoring station (>25 km from the suburban hospital) was adjacent to Detroit (∼2 km from the Wayne County border) and yet, at this location, the annual arithmetic mean PM_2.5_ values for the study period were approximately 25% lower than at the Dearborn monitoring station (http://michigan.gov/documents/deq/deq-aqd-air-2008-Air-Quality-Report_296426_7.pdf); but, with the same seasonal variation. Furthermore, the prevailing winds do not bring pollution from Detroit into this suburban location. Nonetheless, PM exposure represents the largest potential source of misclassification in our study. Another source of potential misclassification involves identification of VTE; however, this bias was minimized because we did not rely on ICD-9-CM codes alone and required confirmation of acute thrombosis from the radiology report. If we had based VTE classification solely on ICD-9-CM codes, 42% of the cases ([1,123+280]/3,310) would have been misclassified. It is possible, however, that some cases designated as ‘chronic DVT’ were in fact acute. Third, our sample size at the suburban hospital was small (n = 417) and so the study may be underpowered to detect seasonal variation there. Fourth, there were differences in group characteristics. For example, the Detroit VTE cases were younger than suburban cases; age may, amongst other effects, affect PM exposure. Similarly, the inter-location differences in the prevalence of the potential risk factors of smoking history, diabetes mellitus, ethnicity, previous VTE, current malignancy, and recent surgery or trauma could also play a role as mentioned in the post-hoc analysis discussion. However, our chi-squared goodness of fit analysis provides some evidence that such factors were not the cause of the summer peak. Fifth, there are limitations when conducting any retrospective chart review, including the accuracy of ICD-9-CM coding and the extent of missing data. There was a considerable amount of the latter in our two no-VTE comparator groups.

### Causality

We emphasize that what we observed is an association between VTE and PM air pollution. The validity of making any etiological inference depends, in part, on study design. For example, the use of ecological studies, where exposure and outcome is measured at the group level, to make inferences on individual outcome is inappropriate [39] and has been termed “ecological fallacy”. However, our study was not an ecological one, but was semi-ecological; the outcome and patient characteristics were measured at the individual level. It has been argued that ecological and semi-ecological studies should be considered different entities and that the inferential properties of semi-ecological studies “do not fundamentally differ from those of individual-level studies” [9]. Therefore, we believe that etiological inferences may be drawn from our findings, especially when considered together with the previously mentioned European and Asian results.Still, are there other potential causes of the observed summer peak in VTE? It is possible that any of the risk factors listed in Table 2 that exhibited a summer peak in their associated VTE cases in Detroit might be responsible for, or at least contribute to, the overall summer peak. But, if that were the case, then we would also expect to find a summer peak for these factors at the suburban location. However, no such peaks were found; for example, there was no difference from a uniform seasonal distribution for diabetes mellitus (P = 0.44), malignancy (P = 0.29), recent surgery or trauma (P = 0.80), or non-idiopathic cases (P = 0.29; all chi-squared goodness-of-fit test). These results are consistent with the concept that there is something specific to Detroit that is responsible for the seasonal variation in VTE. Nevertheless, attribution of causality will require additional study.

### Conclusion

Our finding of periodic variation in VTE with a summer peak is in contrast to previous reports that usually observed winter peaks. Such studies often attributed VTE periodicity to matching periodicity in PM pollution. Our results provide support rather than contradiction for the conclusion of these earlier studies because VTE periodicity in Detroit coincided with the periodicity of specific elements of PM pollution. Our finding of a summer peak appears to eliminate a role for low temperature in the genesis of VTE periodicity. However, attribution of cause to the observed effects is complicated because of the many variables involved and because PM exhibits different regional and seasonal patterns. Conversely, such complexity may permit etiology to be deduced by a process of elimination. Studies to examine VTE occurrence in different locations selected because of their differences in seasonal PM and differences in PM composition may enable the trigger(s) to be identified, or at least allow some variables to be removed from consideration. Finally, we propose that attempts to attribute environmental cause to VTE must incorporate the variable delay between the onset of symptoms and diagnosis.

## Supporting Information

Text S1 **Example calculation of circular statistics parameters.**
(PDF)
