# Supplementary material for: Venous Thromboembolism in an Industrial North American City: Temporal Distribution and Association with Particulate Matter Air Pollution
Source: PLoS One. 2013 Jul 10;8(7):e68829. doi: 10.1371/journal.pone.0068829 (PMC3707887; doi:10.1371/journal.pone.0068829)
Supplement: Text S1 — Example calculation of circular statistics parameters. (PDF) [file pone.0068829.s001.pdf]

Detroit VTE cases

month	M#	DOY	R'DOY	sin'DOY	cos'DOY	#VTEs	sum sin	sum cos	CF	rel. CF	uniform dist	diff
Jan	1	14.5	0.250	0.247	0.969	114	28.16	110.47	114	0.0765	0.0833	-0.0068
Feb	2	45.5	0.783	0.706	0.709	104	73.38	73.70	218	0.1463	0.1667	-0.0204
Mar	3	73.5	1.265	0.954	0.301	119	113.49	35.80	337	0.2262	0.2500	-0.0238
Apr	4	104.5	1.799	0.974	-0.226	139	135.40	-31.43	476	0.3195	0.3333	-0.0139
May	5	134.5	2.315	0.735	-0.678	126	92.66	-85.38	602	0.4040	0.4167	-0.0126
June	6	165.5	2.849	0.288	-0.957	136	39.23	-130.22	738	0.4953	0.5000	-0.0047
July	7	195.5	3.365	-0.222	-0.975	153	-33.96	-149.18	891	0.5980	0.5833	0.0147
Aug	8	226.5	3.899	-0.687	-0.727	137	-94.13	-99.54	1028	0.6899	0.6667	0.0233
Sept	9	257.5	4.433	-0.961	-0.276	111	-106.69	-30.65	1139	0.7644	0.7500	0.0144
Oct	10	287.5	4.949	-0.972	0.235	117	-113.74	27.44	1256	0.8430	0.8333	0.0096
Nov	11	318.5	5.483	-0.718	0.696	131	-94.01	91.23	1387	0.9309	0.9167	0.0142
Dec	12	348.5	5.999	-0.280	0.960	103	-28.86	98.87	1490	1.0000	1.0000	0.0000
						**SUM**	**10.94**	**-88.91**				

DOY – day of year; January 1st = 0 and Dec 31st = 364; the mid-point of each month is used as the DOY

R'DOY – the angle of the DOY expressed in radians; the DOY is first converted to an angle in degrees and then to radians

14.5 days = 14.5 (360/364) degrees = 14.34 degrees = 14.34 (2π/360) radians = 0.250 radians

sin'DOY – the sine of the DOY

cos'DOY – the cosine of the DOY

#VTEs – the number of VTE cases that occurred in each month over the duration of the study

sum sin – sum of the sines for the number of VTE cases in each month; for January, 0.247 * 114 = 28.16

sum cos – sum of the cosines for the number of VTE cases in each month; for January, 0.969 * 114 = 110.47

CF – cumulative frequency of the VTE cases

rel. CF – the relative cumulative frequency; CF/the total number of VTE cases = CF/1490

uniform dist - the expected relative cumulative frequency if the cases were distribution uniformly throughout the year; January = 1/12 = 0.0833

diff – the difference between the relative cumulative frequency and the uniform cumulative frequency

To calculate the mean direction of the distribution, we need the sum of the sine and cosine components as shown in the table as "**SUM**".

R is the resultant vector of the individual vectors and $R^2 = S^2 + C^2$

where S and C are the sums of the sine and cosine components

hence, $R^2 = 10.94^2 + (-88.91)^2 = 8,024.7$

therefore, $R = 89.58$

the angle of the resultant vector, θ = arctan (S/C) = -7.01°

however, because C < 0, we must add 180°; therefore θ = 172.99°

this angle must now be converted to a day. The mean DOY of the distribution = June 24th

To determine the 95% confidence interval of the mean requires the second moment to be calculated

	**DOY**	**R'DOY**	**sin'DOY**	**cos'DOY**	**#VTEs**	**sin2'DOY**	**cos2'DOY**	**sum sin2**	**sum cos2**
Jan	14.5	0.250	0.247	0.969	114	0.479	0.878	54.58	100.09
Feb	45.5	0.783	0.706	0.709	104	1.0000	0.004	104.00	0.45
Mar	73.5	1.265	0.954	0.301	119	0.574	-0.819	68.28	-97.46
Apr	104.5	1.799	0.974	-0.226	139	-0.441	-0.898	-61.23	-124.79
May	134.5	2.315	0.735	-0.678	126	-0.997	-0.082	-125.58	-10.29
June	165.5	2.849	0.288	-0.957	136	-0.552	0.834	-75.13	113.36
July	195.5	3.365	-0.222	-0.975	153	0.433	0.901	66.22	137.93
Aug	226.5	3.899	-0.687	-0.727	137	0.998	0.056	136.79	7.66
Sept	257.5	4.433	-0.961	-0.276	111	0.531	-0.848	58.91	-94.08
Oct	287.5	4.949	-0.972	0.235	117	-0.456	-0.890	-53.34	-104.13
Nov	318.5	5.483	-0.718	0.696	131	-1.000	-0.030	-130.94	-3.94
Dec	348.5	5.999	-0.280	0.960	103	-0.538	0.843	-55.41	86.82
							**SUM**	**-12.87**	**11.62**

The resultant vector of the second moment, $R_2 = \sqrt{(-12.87^2 + 11.62^2)} = 17.34$

The sample's circular dispersion, $\delta = (1 – R_2/n) / [2 (R/n)^2] = 136.7$

The circular standard error, σ, is calculated from $\sigma^2 = \delta/n$ and therefore $\sigma = 0.303$

The 95% confidence interval is given by mean + arcsin (1.96 * σ) and mean – arcsin (1.96 * σ) = mean ± 36.43°

After combining this with the mean angle calculated above and then converting to DOY, the 95% confidence interval is May 19th to July 31st

For Kuiper's test, the test statistic, k, is calculated as $n^{1/2}$ $(D^+ + D^-)$, where n is the sample size and D^+ and D^- are the absolute values of the largest positive and negative differences between the observed relative cumulative frequency distribution and the uniform cumulative frequency distribution. These latter values are shown in the column labeled "**diff**" and, in this case are $D^+ = 0.0233$ and $D^- = 0.0238$.

therefore, $k = 1490^{1/2} (0.0233 + 0.0238) = 1.818$

from published tables, the corresponding *P*-value is between 0.02 and 0.05

For the Rayleigh test, $P = \exp(-Z)$; where $Z = R^2/n$

therefore, $P = 0.0046$

For the periodic regression, a transformation of the data is done; i.e., taking the sine and cosine of the DOY. Then, a multiple regression analysis is performed with X_1 = sin'DOY; X_2 = cos'DOY; Y = VTE cases

The multiple regression equation is of the general form

$Y = a + b_1X_1 + b_2X_2$

a = 124.2 – this is the periodic mean and the values of b are as indicated below.

The values listed as B are the standardized regression weights.

	b	B	B x r_{xy}
X_1	0.533	0.0256	0.0006
X_2	-15.5121	-0.7414	0.5496

Multiple R^2 = 0.5502

Adjusted Multiple R^2 = 0.4503

ANOVA Table

Source	SS	df	MS	F	P
**Regression**	1439.2522	2	719.6261	5.51	0.0274
**Residual**	1176.4145	9	130.7127		
**Total**	2615.6667	11			

Therefore the equation of the periodic regression is, VTE = 124.2 + 0.533 * sin'DOY – 15.512 * cos'DOY

The amplitude of the wave = $\sqrt{(0.533^2 + 15.512^2)} = 15.52$

The phase angle = arctan (X_1/X_2) = -1.97° then, because X_2 is negative, add 180° and convert to DOY

Therefore, phase angle (the peak of the wave) occurs on June 30th

These calculations are described in greater detail in; **Bell KNI** (2008) Analysing cycles in biology & medicine - a practical introducion to circular variables & periodic regression. St. John's, Newfoundland, Canada: Razorbill Press. **Fisher NI** (1995) Statistical analysis of circular data. Cambridge, UK: Cambridge University Press. **Batchelet E** (1981) Circular Statisitics in Biology. London, UK: Academic Press.
